# Oocyte cryopreservation review: outcomes of medical oocyte cryopreservation and planned oocyte cryopreservation

**DOI:** 10.1186/s12958-021-00884-0

**Published:** 2022-01-07

**Authors:** Zachary Walker, Andrea Lanes, Elizabeth Ginsburg

**Affiliations:** Department of Obstetrics and Gynecology, Division of Reproductive Endocrinology and Infertility, Brigham & Women’s Hospital, 75 Francis Street, Boston, MA 02115 USA

**Keywords:** Cost effectiveness, Fertility preservation, Oocyte freezing, Oocyte warm, Oocyte utilization, Planned oocyte cryopreservation, Vitrification

## Abstract

**Background:**

The utilization of oocyte cryopreservation (OC) has become popularized with increasing numbers of reproductive-aged patients desiring to maintain fertility for future family building. OC was initially used for fertility preservation in postmenarchal patients prior to gonadotoxic therapies; however, it is now available to patients to circumvent age-related infertility and other diagnoses associated with early loss of ovarian reserve. The primary aim of this paper is to provide a narrative review of the most recent and robust data on the utilization and outcomes of OC in both patient populations.

**Summary:**

OC results in similar oocyte yield in patients facing gonadotoxic therapies and patients undergoing planned OC. Available data are insufficient to predict the live birth rates or the number of oocytes needed to result in live birth. However, oocyte yield and live birth rates are best among patients < 37.5 years old or with anti-mullerian hormone levels > 1.995 ng/dL, at the time of oocyte retrieval. There is a high ‘no use’ rate (58.9%) in patients using planned OC with 62.5% returning to use frozen oocytes with a spouse. The utilization rate in medical OC patients is < 10%. There is currently no data on the effects of BMI, smoking, or ethnicity on planned OC outcomes.

**Conclusion:**

It is too early to draw any final conclusions on outcomes of OC in medical OC and planned OC; however, preliminary data supports that utilization of OC in both groups result in preservation of fertility and subsequent live births in patients who return to use their cryopreserved eggs. Higher oocyte yield, with fewer ovarian stimulation cycles, and higher live birth rates are seen in patients who seek OC at younger ages, reinforcing the importance of age on fertility preservation. More studies are needed in medical OC and planned OC to help guide counseling and decision-making in patients seeking these services.

## Background

Oocyte cryopreservation (OC) is used in assisted reproductive technology (ART) to attempt to preserve fertility by freezing gametes for potential future use. The first human pregnancy (twins) from a previously cryopreserved oocyte was reported in 1986 [1]. With improved techniques of cryopreservation (i.e., slow-freeze to ultra-rapid vitrification) there have been tremendous improvement in oocyte survival and clinical pregnancy rates [2, 3]. In 2013 the American Society for Reproductive Medicine (ASRM) removed the “experimental” label associated with OC. The American College of Obstetricians and Gynecologist (ACOG) promoted the utilization of OC in 2014 [4]. At that time, a majority of OC was used for patients with upcoming exposure to gonadotoxic therapies including chemotherapy and pelvic radiation, and genetic disorders predisposing them to primary ovarian insufficiency (e.g., Fragile X premutation and monosomy X mosaicism). A 25% increase in the utilization of OC was seen from 2015 to 2016 [5]. In 2018 the ASRM Ethics Committee opinion stated that planned OC for patients wishing to attempt to protect against future infertility due to reproductive aging was ‘ethically permissible’ [6]. ASRM continues to recommend that providers inform patients about the efficacy, safety, benefits and risks, as well as unknown long-term effects on offspring and potential harms that are still not fully understood [6].

Oocyte cryopreservation has increased worldwide [7]. However, reproductive age patients are still inadequately aware of the effects of age on fertility [8–12]. Most patients seeking OC obtain information from online sources [13]. Interestingly, a study of obstetricians and gynecologists revealed that, although they believe discussions of reproductive aging should be discussed with all reproductive aged patients, a majority reported a lack of time or knowledge to counsel patients on fertility preservation [14]. Some opinions hold that patients are “delaying” childbearing due to career aspirations; however, this is actually not the case. Most patients who pursue OC report that the reason is the lack of a suitable partner and a source of protection against future medical issues that may affect fertility [8, 15–17]. It is imperative that healthcare providers be equipped with the knowledge and information to counsel patients regarding reproductive aging and the option of OC.

The purpose of this narrative review on medical OC and planned OC is to compare the most recent available data on outcomes, expectations, and overall recommendations to help guide decision-making and clinical counseling for patients.

## Materials and methods

### Search strategy and study selection

We searched the published articles in PubMed that contained the key words “autologous IVF”, “fertility preservation”, “oocyte cryopreservation”, “elective”, “cancer”, “medical”, or “planned”. We did not include abstracts, conference proceedings, review articles, or case-reports. Studies that included pediatric, adolescent, or transgender patients were excluded. We included randomized controlled trials, systematic reviews or meta-analyses, cohort studies, and case control studies that had full-length manuscripts that were published in English in peer-reviewed journals up to July 2021. We also included studies focusing on medical OC, planned OC, and simulated/hypothetical patient cohorts if part of a cost-effectiveness study. Animal studies, studies without a comparison group (unless addressing topics of utilization of oocytes), inclusion of donor oocytes, utilizing slow-cooling techniques only, articles pertaining to surgical interventions (e.g., ovarian tissue cryopreservation or fertility-sparing surgery), in vitro maturation, frozen embryos only, and studies focused on laboratory-related technical issues were excluded. We screened the titles and abstracts of potentially related and relevant articles based on the above inclusion/exclusion criteria. We included all articles that pertained to utilization of medical OC and/or planned OC. Final inclusion or exclusion decisions were based on examination of the articles in full.

The following data were recorded: author, year of publication, study design, sample size (patients and/ or cycles), stimulation protocol (if stated), mean age at cryopreservation, mean number of oocytes retrieved, mean number of mature oocytes (MIIs) cryopreserved, return/utilization rate, fertilization rate of MIIs, storage duration, clinical pregnancy rate, and ongoing pregnancy/live birth rate.

## Medical oocyte cryopreservation

Initially, OC was primarily used in patients who were going to be exposed to gonadotoxic therapies or radiation for treatment of malignancies. However, indications have expanded, such as preserving supernumerary oocytes for future use in situations where fertilizing all oocytes is not desired, unexpected unavailability of sperm at the time of oocyte retrieval, and planned female-to-male transition [18]. For the purposes of this review, we primarily focused on medical OC that pertained to patients who were diagnosed with cancer and were expected to undergo gonadotoxic therapy.

There have been significant improvements in the management of cancers leading to increased survival rates. Fertility preservation has become integral to the discussion of cancer treatment in reproductive age patients who are at risk of infertility after therapy. One study found that approximately 70% of young female cancer patients were concerned about fertility at the time of diagnosis, and 50% desired to have children after treatment [19]. The risk of premature ovarian failure or diminished ovarian reserve after cancer treatment is dependent on age, chemotherapy used, dosage of chemotherapy agents, and exposure to pelvic radiation. It is of the utmost priority that oncologists speak with their patients about their risks of infertility and to review potential options for fertility preservation prior to initiation of treatment, or refer them for this counseling.

### Outcomes of medical oocyte cryopreservation

There are limited data regarding outcomes of autologous OC in patients with cancer (Table 1). Cobo et al. published a retrospective, observational multicenter study that included patients who underwent planned OC and medical OC from January 2007 to May 2018. They included 1073 patients (1172 cycles) who underwent medical OC. The mean age was 32.3 ± 3.5 years old with 69.9% of patients being ≤35 years old. They primarily used an antagonist plus letrozole protocol for controlled ovarian stimulation; however, they noticed that the antagonist only protocol yielded higher number of retrieved and vitrified oocytes compared to the long GnRH-agonist and the antagonist plus letrozole groups. The mean number of mature oocytes retrieved and vitrified per cycle was 11.4 ± 3.5 and 8.7 ± 2.1, respectively. Only 80 (7.4%) patients returned to used their cryopreserved oocytes with a mean storage time of 4.1 ± 0.9 years. The embryo transfers were done on either day 3 or at blastocyst stage (days 5-7). The overall survival rate and implantation rate was 81.8 and 32.5%, respectively. The clinical pregnancy rate per transfer was 41.4% and the ongoing pregnancy rate per transfer was 31.0%. There were 18 live births within their cohort of medical OC. When factoring in age, they did not notice a statistically significant difference in number of oocytes needed to have a live birth; however, this was most likely due to small sample size. When compared to patients who underwent planned OC, medical OC patients were younger at the age of vitrification, had higher numbers of oocytes retrieved and vitrified per cycle, longer storage times, lower implantation rates, ongoing pregnancy rates, and live birth rates (*p* < 0.05).

**Table 1:** Studies including cycle outcomes of ovarian stimulation protocols in patients undergoing medical oocyte cryopreservation

Author, year (ref)	Type of study	Study population	Sample size	Stimulation protocols	Mean number of oocytes retrieved	Mean number of mature (MII) oocytes cryopreserved
**Cobo et al., 2018** [7]	Retrospective	POC and MOC	6362 patients ● 5289 POC ● 1073 MOC	Antagonist Agonist Antagonist+letrozole	POC: Antagonist: 10.5 ± 7.3^a^ Agonist: 8.8 ± 5.9 Antagonist+letrozole: NA MOC: Antagonist: 13.4 ± 9.5^a,b^ Agonist: 8.3 ± 6.2 Antagonist+letrozole: 11.2 ± 8.1^b^	POC: Antagonist: 8.1 ± 5.8^a^ Agonist: 6.5 ± 4.8 Antagonist+letrozole: NA MOC: Antagonist: 10.3 ± 7.5^a^ Agonist: 5.8 ± 4.6 Antagonist+letrozole: 8.6 ± 6.6
**Kawwass et al., 2019** [20]	Meta-analysis	POC and MOC	29,631 cycles ● 2715 MOC ● 13,626 POC ● 6863 other ● 6427 medically indicated	Antagonist Standard Antagonist Agonist flare Mixed/other^b^	NA	MOC^c^: 81.1% (95% CI 80.1-82.0) POC (ref)^c^: 82.6% (95% CI 80.6-82.7) Other^c^: 82.2% (95% CI 82.2-83.2) Medical^c^: 81.7% (95% CI 80.8-82.6)
**Marklund et al., 2020** [21]	Prospective	BrCA patients undergoing MOC or embryo cryopreservation	610 women (380 cycles)	Antagonist Antagonist+letrozole Conventional start Random start Antagonist+letrozole+GnRHa trigger Antagonist+letrozole+hCG trigger	Antagonist: 12.21 (range, 0-52)^d^ Antag+letrozole: 12.32 (range, 0-55)^d^ Conventional: 12.3 (range, 0-55)^d^ Random: 12.2 (range, 0-52)^d^ GnRHa: 13.66 (range, 0-55)^a,d^ hCG: 11.32 (range, 0-44)^a,d^	Antagonist: 10 (range, 1-27) Antag+letrozole: 9.7 (range, 0-40) Conventional: 10.6 (range, 0-40) Random: 8.97 (range, 0-24) GnRHa: 10.4 (range, 0-40) hCG: 9.1 (range, 0-28)
**De Moraes et al., 2019** [22]	Retrospective	POC and MOC	187 patients ● 164 POC ● 23 MOC	Variable (primarily Antagonist)	Per patient: POC: 13.0 ± 9.1 MOC: 15.6 ± 9.1 Per cycle: POC: 11.4 ± 8 MOC: 13.8 ± 9	Per patient: POC: 11.1 ± 8.2 MOC: 12.7 ± 7.1 Per cycle: POC: 9.7 ± 7 MOC: 11.2 ± 7.2
**Pereira et al., 2017** [23]	Retrospective	Cancer patients undergoing MOC or embryo cryopreservation	341 patients	Antagonist ● GnRHa trigger ● hCG trigger Antagonist+letrozole ● GnRHa trigger ● hCG trigger	Antagonist^c^: ● GnRHa trigger: 14.5 ± 11.4 ● hCG trigger: 12.5 ± 7.8 Antagonist+letrozole^c^: ● GnRHa trigger: 13.1 ± 7.1 ● hCG trigger: 13.6 ± 7.5	Antagonist^c^: ● GnRHa trigger: 13.3 ± 7.9^a^ ● hCG trigger: 9.3 ± 6.0^a^ Antagonist+letrozole^c^: ● GnRHa trigger: 11.8 ± 5.8 ● hCG trigger: 9.9 ± 6.0
**Quinn et al., 2017** [24]	Retrospective	Patients diagnosed recently with BrCA undergoing POC and MOC	589 patients ● 191 BrCA (with and without letrozole) ● 398 POC	Antagonist	POC: 17.0 ± 0.5^a^ BrCA+letrozole: 20.1 ± 1.1^a^ BrCA: 16.6 ± 1.2	POC: 13.2 ± −0.4 BrCA+letrozole: 14.1 ± 0.8 BrCA: 12.2 ± 1.0
**Schon et al., 2017** [25]	Retrospective	POC and MOC	259 cycles ● 129 POC ● 130 MOC	Agonist^a^ Antagonist^a^ Flare^a^	POC: 10 (range, 6-15) MOC: 11 (range, 8-18)	POC: 7 (range, 4-13) MOC: 8 (range, 6-15)
**Specchia et al., 2019** [26]	Retrospective	MOC	244 patients (252 cycles)	Antagonist Antagonist+letrozole	13.5 ± 8.4 (range 0-40) per patient	9.5 ± 6.1 (range 0-24) per patient
**Turan et al., 2018** [27]	Meta-analysis	MOC	2543 patients ● 713 MOC ● 1835 healthy	Variable	NS	NA

*POC* planned oocyte cryopreservation, *MOC* medical oocyte cryopreservation, *NA* not available, *NS* not statistically significant, *BrCA* Breast cancer, *GnRHa* GnRH agonist

^a^*p* < 0.05

^b^Mixed/other stimulation protocol includes Clomid ± FSH, aromatase inhibitors ± FSH, unstimulated cycle, mixed cycle incorporating more than one protocol

^c^Percentage of mature oocytes using Poisson model for predicted number of oocytes retrieved (does not include cancelled cycles or those with 0 eggs retrieved)

^d^Includes oocyte and embryo cryopreservation

Two retrospective studies by Moraes et al. and Schon et al. also compared medical OC to planned OC [22, 25]. Moraes et al. included 23 cancer patients (primarily diagnosed with breast cancer) and 164 non-cancer patients undergoing medical OC and planned OC, respectively. The mean age was 35.13 ± 3.72 years old. Contrary to Cobo et al., Moraes et al. found no statistically significant difference in mean number of oocytes obtained per cycles (11.4 ± 8 non-cancer vs. 13.8 ± 9 cancer) and mean number of frozen mature oocytes per cycle (9.7 ± 7 non-cancer vs. 11.2 ± 7.2 cancer). However, the inability to find a statistically significant difference may have been due to the small sample size.

Schon et al. included 117 patients (130 cycles) undergoing medical OC to 98 patients (129 cycles) undergoing planned OC. Stimulation protocols used included GnRH agonist, GnRH antagonist, or flare. Patients undergoing medical OC were more likely to use an antagonist protocol compared to patients undergoing planned OC (92.9% vs. 77.8%, *p* = 0.003). When adjusting for age, there were no statistically significant differences in cycle parameters (e.g., gonadotropin dosage, estradiol level, number of follicles measuring > 15 mm at time of trigger) between medical OC and planned OC. There was no difference in number of mature oocytes retrieved (7 (range, 4-13) planned OC vs. 8 (range, 6-15) medical OC, *p* = 0.23).

Another study by Specchia et al. [26] investigated 18 years of experience of medical OC at a tertiary care referral center. They included all 244 patients who underwent 252 cycles of medical OC from January 2001 to March 2019 at a single-center. A majority of the patients were diagnosed with breast cancer (59.9%) or Hodgkin’s or non-Hodgkin’s lymphoma (27.4%). The primary stimulation protocol was an antagonist protocol; however, patients received antagonist plus an aromatase inhibitor if they had hormone-dependent breast cancers. The mean age of the cohort was 31.3 ± 6.4 years, which is similar to that of Cobo et al. [7]. The mean number of oocytes retrieved and mature oocytes vitrified per patient were 13.5 ± 8.4 (range, 0-40) and 9.5 ± 6.1 (range, 0-28), respectively. The return rate for use of cryopreserved oocytes was 4.5% with a short mean duration of storage of 3.4 years showing that as of the time of publication a majority of patients who undergo medical OC had not yet returned to utilize their vitrified oocytes. The reasons for this were not investigated, but may have included cancer progression requiring further treatment, spontaneous pregnancy, or lack of a partner. As tamoxifen use for 10 years is recommended for patients with ER positive breast cancer, some delays may have been due to wanting to maximize disease free survival. The clinical pregnancy rate of the 11 patients who returned to use their cryopreserved oocytes was 36.4% per patient (16.7% per transfer) with a total of 2 live births overall. At the conclusion of the study, 95.7% of oocytes retrieved were still in storage.

A prospective study by Marklund et al. investigated the efficacy and safety of controlled ovarian stimulation protocols using GnRH antagonist with or without the addition of letrozole in breast cancer patients undergoing medical OC [21]. This was a prospective, multicenter study from January 1, 1995 to June 30, 2017 at six Swedish fertility programs. Approximately 600 women were enrolled with 468 undergoing fertility preservation. Forty-one percent of those were undergoing medical OC. The mean age of the overall cohort was 32.5 years old (range, 21-42). Marklund et al. compared GnRH antagonist with letrozole (*n* = 224) with GnRH antagonist without letrozole (*n* = 156), conventional start (*n* = 179) versus random start (*n* = 201), and the use of GnRH agonist trigger (*n* = 96) versus hCG trigger (*n* = 128) in patients using a GnRH antagonist protocol with letrozole. They found no statistically significant differences in number of cryopreserved oocytes when using GnRH antagonist with letrozole versus without letrozole (mean number of cryopreserved oocytes: 9.7 with letrozole vs 10 without letrozole, *p* = 0.81) or between conventional start versus random start (mean number of cryopreserved oocytes: 10.6 conventional vs 8.97 random, *p* = 0.067). However, in patients utilizing GnRH antagonist with letrozole and GnRH agonist trigger had a higher number of oocytes retrieved compared to patients with GnRH antagonist with letrozole and hCG trigger (13.66 GnRH agonist trigger vs. 11.32 hCG trigger, *p* = 0.027). This was also seen in a study by Pereira et al. [23] who investigated cancer patients undergoing controlled ovarian stimulation for fertility preservation with GnRH antagonist protocol with or without letrozole and compared cycle outcomes with either use of a GnRH agonist trigger versus hCG trigger. They included 341 patients with an overall mean age of 33.3 ± 5.1 years. The primary cancer diagnosis was breast cancer (75.3%) followed by lymphoma/leukemia (9.7%). They found that using a GnRH agonist trigger resulted in higher number of MII oocytes cryopreserved compared to hCG trigger in patients using letrozole-based protocols (11.8 ± 5.8 GnRH agonist vs. 9.9 ± 6.0 hCG, *p* = 0.04) and those using gonadotropin-only protocols (13.3 ± 7.9 GnRH agonist vs. 9.3 ± 6.0, *p* = 0.02).

Kawwass et al. [20] conducted a national retrospective review using national surveillance data on 29,631 autologous OC cycles from the Society for Assisted Reproductive Technology Clinical Outcomes Reporting System (SART CORS) performed from 2012 to 2016 to compare outcomes of fertility preservation between patients with and without cancer. When compared to planned OC, patients undergoing medical OC were typically < 35 years old, had higher body mass indices (BMI), lived in the South, and underwent antagonist protocols. There was no difference in cancellation or hyperstimulation rates, or oocyte yield (approximately 16 oocytes, 80% maturation rate) between the two groups, as also shown in prior studies [24, 27]. Neither oocyte fertilization rates nor live birth rates were reported.

Lyttle Schumacher et al. did a cost-effectiveness study on OC for cancer patients prior to high and low-risk gonadotoxic therapy [28]. The purpose of their study was to find the live birth rate and cost-effectiveness of fertility preservation with OC compared to expectant management (no-OC) in 25-40 year old cancer patients based on estimated gonadotoxicity treatment 5 years after cancer diagnosis. Their model took into account the type of chemotherapy, the potential development of primary ovarian insufficiency after treatment, and effect of age on fertility decline. Low-risk chemotherapy was equated to therapies such as adriamycin, bleomycin, vinblastine, and dacarbazine for management of Hodgkin lymphoma, while high-risk chemotherapy was equated to conditioning chemotherapeutic regimens used for hematopoietic stem cell transplant for leukemia. When comparing OC to no-OC, the maximum improvement in live birth rate was achieved at 37 years old for low-risk chemotherapy (68% OC vs. 37% no-OC) and at 27 years old for high-risk chemotherapy (66% OC vs. 14% no-OC). The cost per additional live birth in low-risk chemotherapy ranged from $44,645 to $83,424 with the most cost-effective time period at 37 years old. On the other hand, the cost per additional live birth in high-risk chemotherapy ranged from $34,194 to $75,970 with the most cost-effective time period at 25 years old. Despite OC being more costly, it is the most cost-effective strategy to improve live birth rates in patients expecting to undergo low or high-risk chemotherapy within 5 years of cancer diagnosis and who are not considering donor oocytes.

### Summary

Women cryopreserving oocytes for medical indications appear to have similar oocyte yields as women undergoing planned OC. The return rate for patients utilizing medical OC is < 10%. There are still limited data on pregnancy and live birth rates from oocytes cryopreserved for medical indications prior to potentially sterilizing therapy.

## Planned oocyte cryopreservation

The ASRM published an evidence-based guideline on planned OC in 2021 but data are limited. These guidelines reviewed the most recent literature and cautioned against planned OC without appropriate counseling regarding the sparsity of data and unknown sequelae [18]. We included eleven primary studies for this review (Table 2). The main areas of focus were live birth rates, effect of age on OC, and number of cryopreserved oocytes needed to obtain a live birth.

**Table 2:** Baseline details of pivotal studies included in the review for planned oocyte cryopreservation

Author, year (ref)	Type of study	Study population	Sample size	Mean age at cryopreservation in years	Stimulation Protocol	Mean number of mature oocytes (MIIs) cryopreserved
**Blakemore et al., 2021** [29]	Retrospective	≥ 1 POC	231 patients (280 cycles)	38.2 (range 23-45)	Antagonist, LDL, Microflare	< 35: 13.8 35-37: 11.5 38-40: 9.0 41-42: 9.9 > 42: 6.8
**Cobo et al., 2018** [7]	Retrospective	POC and MOC	6362 patients ● 5289 POC (7044 cycles) ● 1073 MOC (1172 cycles)	POC: 37.2 ± 4.9 MOC: 32.3 ± 3.5	Antagonist Agonist Antagonist+letrozole	POC: 7.3 ± 11.3 per cycle MOC: 8.7 ± 2.1 per cycle
**Doyle et al., 2016** [30]	Retrospective	Women undergoing IVF/ICSI for infertility compared to POC + MOC	128 POC + MOC cycles 1283 vitrified/warmed oocytes 2963 fresh ICSI cycles in infertile couples	POC + MOC: 34.9 Fresh: 35.5	Antagonist, Microflare	POC + MOC: 8.0 Fresh: 10.1
**Garcia-Velasco et al., 2013** [31]	Retrospective	POC and MOC	1035 patients (1080 cycles) ● 560 POC (725 cycles) ● 475 MOC (355 cycles)	POC: 36.7 ± 4.2 MOC: 31.9 ± 5.1	Antagonist Antagonist+letrozole	POC: 9.9 per patient MOC: 8.5 per patient
**Goldman et al., 2017** [32]	Retrospective, modeling study	First fresh male-factor and/or tubal factor only autologous ICSI cycles and egg donation cycles	Male factor and or tubal factor (*n* = 466) Egg donation (*n* = 54)	Autologous: NA Donor: 28.5	NA	≤ 35: 13.7 ± 7.5 36: 14.6 ± 7.4 37: 11.8 ± 6.9 38: 8.5 ± 5.7 39: 8.5 ± 4.9 40: 8.3 ± 5.6 41: 9.7 ± 4.2 42: 10.3 ± 6.9 > 42: 6.9 ± 5.6
**Gürtin et al., 2019** [33]	Retrospective	POC and non-POC	129 patients ● 46 POC (64 cycles) ● 83 non-POC (96 cycles)	POC: 37.7 Non-POC: 37.2	NA	POC: 9.3 per cycle Non-POC: 6.1 per cycle POC: 14.0 per patient Non-POC: 11.2 per patient
**Leung et al., 2021** [34]	Retrospective	POC < 38 versus ≥38 years old	921 patients (1265 cycles)	38.1 ± 1.8 (range 34-42)	NA	< 38: 18.4 ± 9.2 ≥38: 15.2 ± 7.7 per patient
**Maslow et al., 2020** [35]	Retrospective	≥ 1 POC	1241 patients (1799 cycles)	35.6 ± 3.26	Antagonist, Microflare	1st cycles (*n* = 1241): ≤ 35: 15.41 ± 9.53 35-37: 12.12 ± 8.26 38-40: 9.75 ± 7.72 41-42: 7.25 ± 6.76 > 42: 6.12 ± 4.73 2nd cycles (*n* = 401): ≤ 35: 10.87 ± 7.74 35-37: 9.60 ± 6.44 38-40: 7.84 ± 4.84 41-42: 8.12 ± 6.90 > 42: 4.30 ± 2.95
**Nagy et al., 2017** [36]	Prospective, phase IV, multicenter, observation registry	First cycles using thaw/warmed cryopreserved (slow freeze & vitrification) oocytes (autologous or donor)	193 patients	Slow freeze: 25.1 ± 2.7 (range 20-31) Vitrified: 26.0 ± 2.8 (range 21-31)	NA	NA
**Wafi et al., 2020** [37]	Retrospective, survey	POC	138 completed surveys	35.7 (range 24-42)	NA	17.6
**Wennberg et al., 2018** [38]	Retrospective	POC	254 patients	36.9 (range 23-43)	NA	7.6

*POC* planned oocyte cryopreservation, *MOC* medical oocyte cryopreservation, *NA* not available, *ICSI* intracytoplasmic sperm injection, *OC* oocyte cryopreservation, *LDL* low-dose luteal

### Live birth rates in planned oocyte cryopreservation

Doyle et al. [30] published a retrospective study which found that 128 cycles undergoing autologous embryo transfer (ET) from warmed vitrified oocytes had lower numbers of MII oocytes inseminated (8.0 vs. 10.1, *p* = .0002) and blastocyst-stage embryo transfers (50.9% vs. 66.1%, *p* < .001) when compared to 2963 cycles using fresh oocytes. There were similar fertilization rates (69.5% vs. 71.7%, *p* > .05) and ongoing pregnancy rates (38.6% vs. 36%, *p* > .05), and higher clinical pregnancy rates and clinical pregnancy losses per clinical pregnancy rate in the vitrified oocyte group compared to the fresh oocyte group. One confounding factor in this study is the inclusion of patients undergoing oocyte cryopreservation due to lack of sperm at time of oocyte retrieval (*N* = 52 cycles by 51 patients). Although male factor infertility was not found to be statistically significant between the two groups within the analysis, the inclusion of these additional patients who did not strictly meet the definition of planned OC introduces bias into the results of the study for outcomes within patients undergoing planned OC.

The 2018 study by Cobo et al. [7] investigated the indication for fertility preservation related to success in IVF cycles after planned OC and for medical fertility preservation. As previously noted, this was a retrospective, observational multicenter study that included 6362 patients treated from January 2007 to May 2018. Of these, 5289 patients undergoing 7044 cycles, had planned OC. One of the main outcomes of this study was live birth rate (Table 3). The mean age of patients who underwent planned OC was 37.2 ± 4.9 with 81.1% of patients having their oocytes vitrified > 35 years old. The mean number of mature oocytes retrieved and vitrified per cycle was 9.6 ± 8.4 and 7.3 ± 11.3, respectively. The survival rate was 83.9% with an implantation rate of 42.6%. The clinical pregnancy rate per transfer was 50.7% and ongoing pregnancy rate per transfer was 39.2%. 115 live births occurred within the planned OC group.

**Table 3:** Summary of cycle outcomes and utilization of included studies in patient who underwent planned oocyte cryopreservation

Author, year (ref)	Mean duration in storage (years)	Return rate (%)	Survival rate (%)	Mean Fertilization rate (%)	Clinical Pregnancy Rate (%)	Ongoing Pregnancy/Live Birth Rate
**Blakemore et al., 2021** [29]	< 35: 8 35-37: 6.6 38-40: 5.3 41-42: 4.9 > 42: 5	< 35: 40 35-37: 44.1 38-40: 36.3 41-42: 33.3 > 42: 25.0	< 35: 72.8 35-37: 77.0 38-40: 73.7 41-42: 66.6 > 42: 78.0	68.8	NA	Live birth (fresh/eSET) < 35: NA/3 35-37: 3/10 38-40: 3/5 41-42: 2/0 > 42: NA/NA
**Cobo et al., 2018** [7]	POC: 2.1 ± 1.6 MOC: 4.1 ± 0.9	POC: 12.1 MOC: 7.4	POC: 83.9 MOC: 81.8	NA	POC: 50.7 per transfer MOC: 41.4 per transfer	POC: 39.2% per transfer MOC: 31.0% per transfer
**Doyle et al., 2016** [30]	Vitrified: 8.0 Fresh: 0	Vitrified: NA Fresh: NA	Vitrified: 86.1 Fresh: NA	Vitrified: 69.5 Fresh: 71.7	Vitrified: 54.4 per cycle Fresh: 45.1 per cycle	Vitrified: 38.6% Fresh: 36.0%
**Fuchs Weizman et al., 2020** [39]	1.8-4.8	12.1-15	85%	66-84%	39-84%	NA
**Garcia-Velasco et al, 2013** [31]	POC: 1.7 ± 0.6 MOC: NA	POC: 4.6 MOC: 0.8	POC: 84.8% MOC: NA	NA	POC: 42.3 per patient MOC: 25 per patient	POC: 30.7% per patient MOC: 25% per patient
**Gürtin et al., 2019** [33]	POC: 4.8 Non-POC: 0.4	NA	NA	NA	NA	POC: 17.4% Non-POC: 22.9%
**Leung et al., 2021** [34]	< 38: 4.1 ≥38: 3.2	< 38: 5.6 ≥38: 11.9	Vitrified: 84.9% Slow: 57.1%	< 38: 78 ≥38: 70	< 38: 54.5 (95% CI 37.6-71.5) per transfer ≥38: 39.3 (95% CI 21.2-57.4) per transfer < 38: 64.0 (95% CI 45.2-82.8) per patient ≥38: 52.4 (95% CI 31.0-73.7) per patient	< 38: 48.5% (95% CI 31.4-65.5) per transfer ≥38: 28.6% (95% CI 11.8-45.3) per transfer < 38: 56.0% (95% CI 36.5-75.5) per patient ≥38: 38.1% (95% CI 17.3-58.9) per patient
**Wennberg et al., 2018** [38]	4.0	15	78	62	NA	36-37: 56% per transfer 38-39: 17% per transfer ≥40: 0% per transfer 36-37: 63% per woman 38-39: 26% per woman ≥40: 0% per woman

*NA* Not available, *eSET* euploid single embryo transfer, *POC* planned oocyte cryopreservation, *MOC* medical oocyte cryopreservation

A recent 2021 study by Leung et al. investigated the clinical experience of patients who had undergone planned OC [34]. They performed a retrospective, observational study that included 921 patients (1265 cycles) who underwent planned OC from June 2006 to October 2020 at a single institution within an insurance mandated state. They split their cohort into two groups: < 38 and ≥ 38 years old. Mean age of patients included was 36.6 years with the mean age at time of use at 38.1 ± 1.8 years (range, 34-42). The mean number of mature oocytes vitrified and mean number of cycles used per patient were 17.1 ± 8.6 and 1.4 ± 0.6, respectively. There was no significant difference in mean number of oocytes cryopreserved based on age. Oocyte survival rate was 84.9% after vitrification and 57.1% after slow-freeze. Fertilization rate was 74% among both groups. The clinical pregnancy rate per transfer (54.5% for < 38; 39.3% for ≥38 years old) or per patient who achieved transfer (64.0% for < 38; 52.4% for ≥38 years old) was not different between groups. Also, the live birth rate per transfer (48.5% for < 38; 28.6% for ≥38 years old) or per patient who achieved transfer (56.0% for < 38 year old; 38.1% for ≥38 years old) was not different between both groups. In addition, the live birth rate among all patients who initiated a thaw/warm cycle (*n* = 68) was 38.9% for < 38 years old and 25% for ≥38 years old. No successful pregnancy occurred in patients who utilized planned OC when ≥40 years old. Some limitations of this study were the inclusion of slow-freeze and vitrification cycles and the limited generalizability given that the study was conducted in an insurance mandated state.

Blakemore et al. conducted a large retrospective study at a single, urban university-affiliated fertility center on patients who underwent ≥1 cycle of planned OC from January 2005 to December 2009 [29]. They analyzed 231 patients (280 cycles). Eighty-eight patients (38.1%) returned to thaw/warm oocytes for intended intracytoplasmic sperm injection (ICSI) and embryo transfer. The mean age of patients returning to thaw/warm oocytes was 43.9 years (range, 38-50 years) with a mean duration of oocyte vitrification of 5.9 years (range, 1-12 years). The mean survival rate post-thaw was 74.2% (median 73.7%, range 0-100%) and the mean fertilization rate was 68.8%; these rates did not differ based on age (*p* = 0.79). There were 41 fresh ET of thawed/warmed oocytes. Thirteen embryo transfers (31.7%) with a mean of 3.4 embryos transferred were performed on Day 3. The remaining 28 transfers (68.3%) with a mean number of embryos transferred of 1.8 were performed on day 5. Of those who underwent a fresh transfer, 28.2% of patients achieved a live birth. In addition, 49 patients underwent preimplantation genetic testing for aneuploidy (PGT-A) with the mean of 4.2 embryos being biopsied per patient (range, 0-14). The average euploidy rate was 28.9%. The live birth rate per transfer of an euploid embryo was 66.7% within the PGT-A group.

A study by Wennberg et al. [38] on planned OC included 254 patients who underwent planned OC at a private IVF center in Sweden between August 1, 2011 to August 31, 2017. The mean age at first vitrification was 36.9 (range, 23-43) and the mean number of frozen oocytes per patient undergoing ≥1 retrieval was 7.6 (range, 1-37). The mean age of those who utilized their oocytes was 38.7 (range, 36-42) at vitrification and 42.7 (range, 38-45) at thaw/warming. Within the group of patients who returned to use their oocytes, the mean number of mature oocytes banked was 12.8 (range, 1-37) with a 78% survival rate and 62% fertilization rate. Live birth rates per transfer were 56, 17, and 0% at 36-37, 38-39, and ≥ 40 at age of vitrification, respectively. Alternatively, the live birth rate per patient was 63, 26, and 0% at 36-37, 38-39, and ≥ 40 at age of vitrification, respectively. One interesting characteristic of this study worth highlighting is that the local recommendation for patients undergoing planned OC was to obtain ≥15-20 oocytes to achieve an live birth; however, only 18% of patients were able to achieve this goal suggesting that live births are still possible with lower oocyte yields.

Gürtin et al. performed a retrospective study of planned OC within the UK over a 10-year time frame [33]. They sought to understand more the characteristics of the group of patients who returned to use their eggs. This analysis included 129 patients stratified into two categories: social egg freeze (SEF) and non-SEF. Non-SEF incorporated egg freeze for clinical reasons (e.g., intentional part of IVF treatment or to batch eggs), incidental egg freeze (e.g., no sperm available at time of egg retrieval), and ethical egg freeze (e.g., patients who did not believe in freezing embryos). Ultimately 46 patients underwent 64 cycles in the SEF group (planned OC). The average age at time of freeze was 37.7 years within the SEF group. The success rate, which was defined as both live birth and ongoing pregnancy, was 17.4% within the SEF group.

A 2020 retrospective questionnaire of patients who had previously undergone planned OC between January 2009 and September 2016 at a single center within the UK also reported on live birth rates [37]. Three hundred and forty-two patients had undergone planned OC during this time frame; however, only 138 completed surveys. The mean age at vitrification was 35.7 (range, 24-42) and the mean follow-up was 4.5 ± 2.4 years. The mean number of oocytes cryopreserved was 17.6 (range, 2-64). Sixty-one patients reported they tried to conceive after planned OC. Forty-six percent (28/61) of respondents reported that they used their cryopreserved eggs to try to conceive and 46% (13/28) reported having a live birth. The number of ET performed was not reported.

Two smaller observational studies done by Garcia-Velasco et al. and Nagy et al. also investigated ongoing pregnancy and live birth rates. Garcia-Velasco et al. reported the results of controlled ovarian hyperstimulation in oncological and non-oncological patients seeking fertility preservation. Five hundred and sixty non-oncological patients were included in the study (mean age 36.7 ± 4.2 years); 26 patients returned for thaw/warming of frozen oocytes. Twenty-four fresh embryo transfers were performed (mean number of embryos transferred 1.5 ± 0.6). The clinical pregnancy rate per patient was 42.3% and the ongoing pregnancy was 30.7% after a fresh embryo transfer [31]. Fifteen patients underwent a frozen embryo transfer (mean number of embryos transferred 2.3 ± 0.7), and had a clinical pregnancy rate per patient of 46.6% and ongoing pregnancy rate per patient of 33.3% [31]. Nagy et al. reported ART outcomes after autologous oocyte vitrification and thawing in 46 cycles after planned OC (mean age 33.9 ± 3.9 years old (range, 25-43 years)) [36]. The mean number of embryos transferred per cycle was 2.7 ± 1.0. (3.0 ± 1.0 day 3 vs. 2.2 ± 0.7 day 5/6). Twelve transfers (30%) resulted in a clinical pregnancy and 8 cycles resulted in a live birth (17.4%). Unfortunately, neither investigation used a control group.

A retrospective cohort study, by Maslow et al., of 1241 patients (1799 cycles) at a single, large OC program revealed the likelihood to achieve an estimated live birth rate (eLBR) of 50, 60, and 70% within 1-2 cycles of planned OC [35]. Using data from Doyle et al. and Goldman et al. [30, 32], they extrapolated the thresholds for an age-based number of frozen mature oocytes needed to obtain a 50, 60, and 70% eLBR. The mean number of frozen MIIs based on age is included in Table 2. They found that the main contributors to success in this study were young age, high AMH, high peak estradiol, and low total gonadotropin usage. Sixty-six percent of patients in this study achieved a 50% eLBR with their first cycle while 51% of patients achieved an eLBR of 70% with their first cycle. Having an AMH > 1.995 ng/dL was predictive of an eLBR of 60% with their first cycle regardless of age (*p* < 0.001). In addition, patients < 37.5 years old were more likely to obtain a 60% eLBR with their first cycle regardless of AMH level (*p* < 0.001). For patients who did not reach a 50% eLBR during their first cycle, 69.3% were able to achieve at least 50% eLBR during their second cycle. Patients > 42 years old were not able to reach 50% eLBR within two cycles. Maslow et al. were able to generate a table from their data using age, AMH level, and number of frozen MIIs to predict eLBR (Table 4).

**Table 4:**
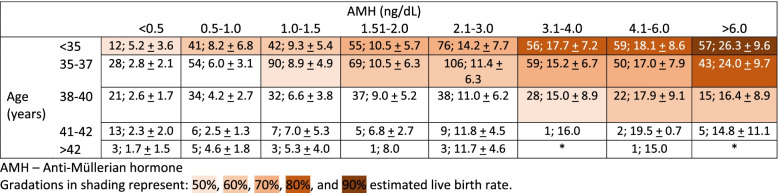
Maslow et al. number of frozen MII oocytes in first retrieval by age and AMH, presented as n; mean ± SD

### Effect of age at time of oocyte retrieval

There is currently insufficient data to counsel patients on the optimal age at which to undergo planned OC. Not surprisingly, the few studies available on this subject indicate that younger oocyte age results in higher live birth rates per embryo transfer.

Doyle et al. [30] found that patients who undergo autologous planned OC < 38 years old had higher clinical pregnancy rates per embryo transfer than patients ≥38 years old at time OC (60.2% vs. 43.9%). However, the data included patients who underwent planned OC as well as patients who underwent OC for other indications. Nagy et al. [36] found that live birth rates among patients who underwent planned OC at < 35 years old were significantly better than patients ≥35 years old (24 (23.8%) vs. 26 (12.0%), *p* < .05); however, this study had several limitations because they used the Human Oocyte Preservation Experience registry to enroll patients. Limitations that are accompanied with use of a registry include selection bias due to entry of non-sequential patients, missing data, changes in cryopreservation technique, small sample size, and the inclusion of patients that underwent medically indicated OC due to lack of sperm at time of egg retrieval. Gürtin et al. [33] also noticed a decline in successful outcomes (defined as live birth rate and ongoing pregnancy rate) with increasing age at time of first vitrification; however, their numbers were too small to measure statistically. Cobo et al. [7] investigated the probability of live birth based on the age at vitrification and found significantly higher cumulative live birth rates for younger patients (≤35 years old) compared to older patients (> 35 years old) (*p* < 0.0001).

Leung et al. [34] reported contrary findings on the significance of age at the time of vitrification. The mean age at time of vitrification was 38.1 ± 1.8 years old (range, 34-42 years) with the mean age at oocyte thaw of 41.8 ± 2.1 years old. They did not find any statistical significance in cumulative live birth rates between patients who vitrified oocytes at < 38 and ≥ 38 years old most likely due to their low return rate (7.4%) and subsequent small sample size (*n* = 46) (Table 3). However, no patients who vitrified ≥40 years old had a successful pregnancy.

A systematic review and meta-analysis done by Fuchs Weizman et al. included 43 studies and found that planned OC is the most cost efficient at 35 years old (assuming a minimum of 60% utilization) [39, 40]. Another study by Mesen et al. found that planned OC was cost-effective at 37 years old if patients were willing to use donor sperm [41]. Mesen et al. used two separate models to perform a cost-effectiveness analysis based on the time a patient makes the decision to undergo planned OC to attempting conception 3, 5, or 7 years later. Model A compared OC to no-OC and assumed that a patient would only attempt to conceive after marriage. Model B compared OC to no-OC and assumed that a patient would attempt to conceive regardless of marital status (willing to use spouse, partner, or donor sperm). Model A patients who used OC were not found to have a substantially better live birth rate (< 10%) when compared to Model A patients who did not undergo planned OC and were awaiting marital status. Model B was found to be the most cost-effective if planned OC was performed at 37 years old compared to other Model B patients who did not undergo planned OC and were not awaiting marital status. The probability of live birth rate was 29.7% higher (51.6% live birth rate for OC vs. 21.9% live birth rate for no-OC) and approximately $9000 more expensive in cost ($19,493 with OC vs. $10,943 without OC) [41].

Another study by Devine et al. found that it was more cost-effective for women who were > 38 years old to defer planned OC and to undergo two cycles of IVF [42]. Devine et al. used theoretical models to map out three different strategies for 35 year old patients who deferred childbearing for personal reasons until 40 years old. Strategy 1 (OC) consisted of undergoing 1.2 cycles of planned OC (mean number required to obtain 16 MII oocytes at 35 years old), attempt spontaneous conception at 40 years old for 6 months, 2 oocyte thaw cycles using stored oocytes if no live birth. Strategy 2 (OC/IVF) consisted of undergoing 1.2 cycles of planned OC (mean number required to obtain 16 MII oocytes at 35 years old), attempt spontaneous conception at 40 years old for 6 months, 2 fresh autologous ART cycles if no live birth, 2 oocyte thaw cycles using stored oocytes if still no live birth. Strategy 3 (no-OC) consisted of no OC at 35 years old, attempt spontaneous conception at 40 years old for 6 months, 2 fresh autologous ART cycles if no live birth. Strategy 1 was found to be the most cost-effective in achieving live birth until 38 years of age when Strategy 3 was most cost-effective, thereby presuming it may be more cost-effective for patients who are considering to undergo planned OC at ≥38 years old to try spontaneous conception for 6 months then if not pregnant to undergo 2 fresh autologous ART cycles.

### How many oocytes should be frozen to have a reasonable chance at live birth?

Few studies have evaluated the number of cryopreserved mature oocytes needed to achieve a live birth. Based on their data, Doyle et al. estimated that to achieve a 70% chance of one live birth in their cohort of 128 autologous thawed/warmed treatment cycles, patients 30-34 years old would need 14 MIIs, 35-37 years old would need 15 MIIs, and 38-40 years old would need 26 MIIs [30]. Cobo et al. found that 10 or 15 vitrified oocytes yielded success rates of 42.8% (95% CI = 31.7-53.9) and 69.85% (95% CI = 57.4-82.2) for cumulative live birth rates, respectively. For women ≤35 years old, the cumulative live birth rate plateaued at 24 vitrified oocytes with a 94.4% [95% CI = 84.3-100.4] cumulative live birth rate.

Goldman et al. conducted a modeling study from a retrospective analysis of their academic infertility center data. They included 520 fresh autologous cycles using ICSI in patients with only male factor infertility, tubal factor infertility, and/or egg donation. Patients with evidence of hydrosalpinx, diminished ovarian reserve, or utilizing preimplantation genetic testing were excluded from the study. Statistical models that included the percentage of mature eggs expected to survive warming, fertilized and become blastocysts were developed to calculate blastulation potential per mature oocyte retrieved. The probability of any one blastocyst being euploid was calculated using age-specific data from Reprogenetics. They included data of 520 patients and found that patients 34, 37, and 42 years old would have to freeze 10, 20, and 61 MIIs, respectively, to achieve a 75% chance of having one or more live birth [32]. The data were used to develop a phone app that can be used to calculate expected live birth rates at different ages based on the number of MII eggs frozen [43].

Nagy et al. employed a similar concept using autologous planned OC treatment cycles and found that patients < 35 years old required 38.8 MIIs to achieve one live birth while patients ≥35 years old required 77 MIIs [36]. However, their data included a small sample size for planned OC, nonelective indications for cryopreservation, and conclusions based on calculated values and not actual data.

The ASRM practice committee concluded that there is insufficient evidence to counsel patients on the ideal number of oocytes required to achieve one live birth with planned OC [18].

### Utilization of cryopreserved oocytes

The 2018 study by Cobo et al. also investigated utilization of cryopreserved oocytes [7]. They noticed an increase in the utilization of planned OC from 4 to 22% over the course of the study, from 2007 to 2018. The return rate for patients who used planned OC was 12.1% with a mean storage time of 2.1 ± 1.6 years.

Blakemore et al. studied a 10-15 year follow-up period after planned OC [29]. Of the 231 patients enrolled only 88 (38.1%) of patients underwent thaw/warming while 136 (58.9%) did not use and 7 (3.0%) transported oocytes to another center with unknown outcome. Rates of utilization did not differ by age group. They also found that of the 88 patients who utilized their vitrified oocytes most returned to thaw/warm oocytes with a partner (*n* = 55, 62.5%) compared to donor sperm (*n* = 33, 37.5%) and this did not differ based on age. Fuchs Weizman et al. also evaluated the utilization of oocytes after planned OC in a systematic review and found that 12.1-15% of oocytes were used 22-58 months after freezing [39].

A recent 2021 study by Leung et al. found a lower return rate of 7.4% over a 14 year time-frame [34]. Patients who were ≥ 38 years old at time of vitrification were more likely to utilize their cryopreserved oocytes (11.9%) compared to patients < 38 years old (5.6%)(*p* < 0.0009). The average storage duration was 3.7 ± 1.7 years. Most patients who returned to thaw/warm their oocytes had a partner to provide sperm, which sperm source (e.g., partner vs. donor) was not statistically significant between groups. The authors did identify that the mean age of first freeze is steadily declining within recent years; however, individuals who have used their frozen eggs have been in older age groups (≥38 years old).

Wafi et al. [37] found that 65% of survey respondents who had undergone planned OC anticipated using their oocytes in the future, and the maximum age considered for use of frozen eggs was 45.2 years old. 98% of respondents reported that they would recommend planned OC to others. Interestingly, when asked about what they would do about unused cryopreserved oocytes, 62% reported donating them to research and 14% would donate them to other patients.

### Obstetrical & Perinatal Outcomes

There are limited studies on the obstetrical and perinatal outcomes of babies born after planned OC. The limited amount of evidence available conclude that neonatal outcomes are similar between fresh and cryopreserved oocytes in infertile patients [31, 44–47].

### Effects of BMI, ethnicity, and smoking on planned OC

There are currently no data available investigating the effects of demographics on planned OC (e.g., ethnicity, body mass index, smoking). Future studies are needed to uncover the potential impact of these factors that may ultimately affect decision-making and counseling.

### Summary

Planned OC is a valuable option for patients wishing to attempt to preserve their future fertility. Available data are sparse, so how many cryopreserved oocytes lead to a high likelihood of live birth is still not concretely defined. As expected, due to the impact of age on ovarian reserve and oocyte aneuploidy, patients who undergo planned OC at younger ages have the highest likelihood and potentially require the least number of cycles. There does not appear to be an increase in congenital anomalies with use of planned OC.

Thus far it appears that most patients who undergo planned OC do not return to use them, regardless of age at time of preservation [29]. Surveys of patients who have undergone planned OC and achieved natural conception do not regret undergoing planned OC [16]. There is currently a strong push by Society for Assisted Reproductive Technology (SART) and ASRM to monitor and report planned OC cycles to accrue more data and help guide management options for future patients [18].

## Conclusion

It is too early to draw any final conclusions on outcomes of OC in medical OC and planned OC; however, preliminary data supports that utilization of OC in both groups result in preservation of fertility and subsequent live births in patients who return to use their cryopreserved eggs. Higher oocyte yield, with fewer ovarian stimulation cycles, and higher live birth rates are seen in patients who seek OC at younger ages, reinforcing the importance of age on fertility preservation. There is currently a low utilization rate of cryopreserved oocytes within each group. Less than 10% of medical OC patients and approximately 40% of planned OC patients have returned to use their cryopreserved oocytes, which limits future studies on clinical outcomes. More studies are needed in medical OC and planned OC to help guide counseling and decision-making in patients seeking these services.

## Data Availability

Data sharing not applicable to this article as no datasets were generated or analyzed during the current study.

## Abbreviations

ACOG: American College of Obstetricians and Gynecologist
ART: Assisted Reproductive Technology
ASRM: American Society of Reproductive Medicine
BMI: Body mass index
eLBR: Estimated live birth rate
ET: Embryo transfer
ICSI: Intracytoplasmic sperm injection
IVF: In vitro fertilization
MII: Metaphase II/mature oocyte
OC: Oocyte cryopreservation
PGT-A: Preimplantation genetic testing for aneuploidy
SART CORS: Society for Assisted Reproductive Technology Clinical Outcomes Reporting System
SEF: Social egg freeze
